# Blood Lead Levels among Children in Yaoundé Cameroon

**DOI:** 10.3389/fpubh.2017.00163

**Published:** 2017-07-07

**Authors:** Francisca Monebenimp, Gilbert Kuepouo, David Chelo, Pieme Constant Anatole, Anne-Cécile Zoung Kany Bissek, Perry Gottesfeld

**Affiliations:** ^1^Faculty of Medicine and Biomédical Sciences (FMBS), Department of Public Health, Service of Pediatrics, University Teaching Hospital of Yaoundé, Yaoundé, Cameroon; ^2^Centre de Recherche et d’Education pour le Développement (CREPD), Yaoundé, Cameroon; ^3^Occupational Knowledge International, San Francisco, CA, United States

**Keywords:** lead poisoning, Africa, blood lead level, lead exposure, Cameroon

## Abstract

Blood lead levels (BLLs) are a useful indication of a population exposure to lead from environmental sources. No previous published study had reported BLLs in Cameroon. Our objective is to characterize exposure levels in children to inform policymakers of potential lead exposure sources. We tested the BLLs of 147 children aged 12 months to 6 years residing in Yaoundé, Cameroon, and conducted an extensive questionnaire with their parents or guardians to characterize potential exposure sources. The geometric mean BLL among this population was 8.0 μg/dl and arithmetic mean level was 8.7 μg/dl. These levels are more than sixfold higher than the geometric mean BLL reported in the U.S. and more than fivefold higher than those reported in France. In addition, 88% of the children tested had lead levels greater than 5 μg/dl. One limitation of the study is that the selection of the children sampled was not a random survey. The analysis of the responses to the questionnaire failed to uncover any specific exposure patterns. A statistically significant association was noted between the age of the child’s home and the duration of exclusive breastfeeding with BLLs. The study points to a need for greater efforts to control sources of lead exposure in Cameroon.

## Introduction

There are many known sources of lead exposure in Cameroon and throughout Africa that are contributing to children’s lead burden. In Cameroon, lead paint is sold in retail outlets, although some companies have removed lead paint additives in recent years. Locally made aluminum cookware has also been shown to leach significant concentrations of lead during normal cooking (1). Local food including spices and tubers contain significant background levels of lead (2). Lead emissions from improper lead battery recycling, industrial sources, combustion products, lead-containing products, and the legacy of lead additives in gasoline have contributed to environmental exposures.

Although lead was banned from gasoline in Cameroon in 2004, it is likely that soil contamination persists in urban areas based on experience in other countries following this action. Exposures from this source in combination with lead from paint, dust, air, food, and water contribute to children body burden. Take-home exposures from parents occupationally exposed have also been responsible for lead poisoning cases in the past in many countries and are an ongoing risk factor that must be considered in Cameroon.

Lead absorption is known to increase with poor nutrition and even missing meals is an important consideration in evaluating potential lead exposures. In South Africa, a country with a significantly higher per capita income than Cameroon, 77% of urban households and 72% in rural areas reported skipping or reducing the size of meals (3). Although similar data are not available for Cameroon, we expect that nutritional status is lower than in South Africa.

Until now there have been no published studies documenting blood lead levels (BLLs) in Cameroon and no laboratory in the country is performing these tests on a routine basis. This report is the first such attempt to assess and characterize BLLs among children aged 12 months to 6 years residing in the nation’s capital city of Yaoundé.

## Materials and Methods

Ethical approval was obtained from the National Ethic Committee of Research for Human Health on July 31, 2015, as well as the administrative authorization issued by the Minister of Public of Cameroon. We recruited parents and guardians of 147 children aged 12 months to 6 years to participate from a convenience sample stratified across a number of geographically diverse neighborhoods. Children were recruited in the town of Yaoundé from the councils of Yaoundé V (Mvog Ada), Yaoundé II (MokoloElobi and Nkongkana), and Yaoundé VI (BiyemAssi) during October and November of 2015. District officers, traditional chiefs of the quarters, mayors’ assistants responsible for health matters, and volunteer community members helped the project team in recruiting children in each locality.

The parent or guardian accompanying each child was briefed on study protocols, and we obtained a signed consent form for their children to participate. A questionnaire was administered to assess potential sources of lead exposure and other potential risk factors. Information was collected on parents’ occupations, potential exposure sources, housing, breastfeeding practices, and children’s development.

The interviews with parents or guardians indicated that 60% of the participants own their homes or apartments. The mean age of the participants’ homes was 24 years old with the oldest structures reported to be approximately 60 years old. 56% of the participants reported that the paint on the interior or exterior of their homes were in poor condition. It is noteworthy that 100% of the participants indicated that they cook with artisanal aluminum cookware. Approximately 53% of the parents had attended either secondary school or university and 21% reported that they smoke.

Collection of venipuncture blood sample was conducted after careful cleaning of the skin at the puncture site. Subsamples were analyzed for Lead using the LeadCare^®^ Ultra™ Blood Lead Testing System to quantify the concentration of lead in a whole blood. The LeadCare device is an *in vitro* diagnostic device that utilizes Anodic Stripping Voltammetry and a sensor to detect lead in whole blood. The analyzer measures the amount of lead on the Sensor and displays the result in micrograms per deciliter.

The LeadCare has a lower limit of detection (LOD) of 3.3 μg/dl. Values below the LOD can be used for statistical analysis to estimate the mean BLL for the study population (4). All the BLL results below the instrument detection level of 3.3 were recorded as 3.2 μg/dl for the statistical summary. Given that only two readings in this study (1%) were below the instrument LOD, these results are unlikely to influence the reported mean. The LeadCare has a built-in electronic calibration system, and prior to being used in testing, the equipment underwent satisfactory calibration procedures and performance verification following the Manufacturer’s operation manual. Calibration was repeated every time we started a new lot of test kits.

Samples of blood collected in the field were transported to the laboratory for analysis. Testing for a complete blood count and hemoglobin was performed by the laboratory of the hematology unit at the University teaching hospital of the University of Yaoundé I. The Mindray BC-5300 equipment from Shenzen Mindray Biomedical Electronics Co, Ltd. was used for this purpose. Subsamples from these same vials were then used to test for iron at the Laboratory of the Department of Biochemistry, at the University of Yaoundé I.

Data from the questionnaire and blood test results were codified to maintain medical confidentiality and entered into an Excel spreadsheet. The data were then provided to Ramboll Environmental (USA) for statistical analysis. Univariate analysis was conducted to assess the distribution of continuous variables for possible associations with BLLs.

A multivariate analysis with key parameters including the reported age of the home, home ownership, presence of bare soil, and the breastfeeding removal age, among other factors, was performed. Breastfeeding removal age was found to be significant (*p* < 0.05) in the overall model. However, there were a large number of missing data from volunteers who were unable to report on several variables that at times influenced these relationships (e.g., age of the home was not significant but influenced the bare soil variable). We then conducted an analysis to assess the impact of the missing cases on the multivariate model and found that the influence of these missing cases was significant. Based on this, we concluded that the reduced sample size (*n* = 81) influenced the associations observed in the overall model and the significance of the findings may have been an artifact of the missing cases. Given our lack of confidence in the results from the multivariate analysis, we did not report these values.

## Results

**Figure 1:**
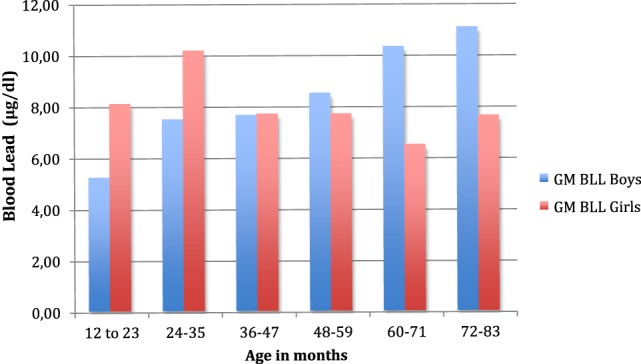
Geometric mean blood lead levels (in μg/dl) vs. age (in months) of the participants to the study. Blue color plots of the histogram show the geometric mean blood levels for boys peaking at around age 6, and red color plots of the histogram show the geometric mean blood lead levels for girls peaking at around age 2.

The geometric mean BLL in the study population was 8.0 μg/dl and the arithmetic mean was 8.7 μg/dl. Results ranged from <3.3 to 25.1 μg/dl. Although boys and girls tested had very consistent geometric mean BLLs (see Table 1), the age distribution showed significant differences. Figure 1 shows BLLs among girls peaking at around the age of 2 years, whereas boys showed increasing levels through the age of 6 years (see Table 2). Note that the study population included more than three times the number of girls than boys (ratio 114:33).

**Table 1:** Blood lead levels by gender.

Gender	*N*	Geometric mean (μg/dl)
Male	33	8.0
Female	114	8.0
Overall	147	8.0

**Table 2:** Blood lead levels (BLLs) by child’s age.

Childs age (months)	*N*	BLL (μg/dl)	SD
12–23	17	7.1	4.5
24–35	25	9.8	5.2
36–47	35	7.7	2.7
48–59	34	7.8	2.9
60–71	18	7.0	2.0
72–83	18	8.4	5.0
Total/geometric mean	147	8.0	3.9

Results indicate that 88% of the children tested had lead levels greater than 5 μg/dl and 32% exceeded 10 μg/dl. Only two of the 147 children tested had BLLs below the instrument detection level of 3.3.

A univariate analysis of the BLL results matched with responses provided by parents and guardians on the questionnaire (see Table 2) noted a statically significant association with the reported age of the child’s home (*p* < 0.01) (see Table 3). However, there were 41 (28%) participants that did not know the age of their home as they were not the owners.

**Table 3:** Univariate analysis of blood lead levels.

Variable	*n*	Ln β	e^(Ln β)^	*p*-Value
Age of home	106	0.01	1.01	0.004*
Age of child	147	−0.002	1.00	0.45
Distance from road	128	0.00	1.00	0.76
Child’s weight	146	−0.01	0.99	0.31
Breastfeeding removal age	112	0.02	1.02	0.01*

*Statistical significance (*p* ≤ 0.05).

Several questions on the survey questionnaire addressed breastfeeding. The age when children stopped breastfeeding was positively associated with higher BLLs. The reported age that children were weaned and stopped taking breast milk was statistically significant (*p* = 0.01) (see Table 3). Other self-reported variables including the distance of the home from the closest road, and the age and weight of the child were not associated with BLL in this analysis.

## Discussion

Socioeconomic status of participating families was not investigated because any cursory attempt to quantify economic status would likely be biased based on the response of the parent or caretaker and not necessarily reflect the household income.

Aside from the age and gender related differences noted, exposures appear to be fairly consistent among the study population. The lack of significant correlations from responses to the questionnaire (other than those reported) suggests that consistent exposure sources may be present among the population studied.

The results from this investigation are generally consistent with recent reports of BLLs reported in other African countries. Mean BLL reported among children in Nigeria were 8.7 μg/dl (5). The geometric mean BLL among children (age < 6 years) reported in Kinshasa, Democratic Republic of Congo was 11.5 μg/dl (6). Mean BLLs reported in urban areas of South Africa were 7.9 μg/dl (7).

The mean BLL among children (12–24 months) reported in a semirural area in Benin were 7.2 μg/dl (8). The study also found that the presence of paint chips in the home was associated with higher BLLs and that maternal BLLs were highly correlated with their children’s level.

Our data suggest that children with longer duration of exclusive breastfeeding may have higher BLLs. Other studies have identified breast milk as a significant source of exposure to infants and suggest that the transfer of lead to breast milk may be higher at lower exposure levels (9). However, the link to breastfeeding noted in our report should be considered preliminary although it may warrant further investigation. The great majority of women in the study population reported breastfeeding and no effort was made to control for other possible risk factors.

Cameroon discontinued the use of lead in gasoline in 2004 but other sources of exposure are known to be present (10). In recent years, it was discovered that much of the paint being sold in stores for architectural applications contained lead in significant concentrations (11). The study found that 66 percent of new paints contained lead at concentrations exceeding 90 parts per million (ppm) and median concentration was 2,150 ppm. Although subsequent surveys conducted since then have demonstrated that the largest brand has since reformulated and removed lead additives from its products, some other domestic and most of the imported brands still contain significant amount of lead.

Homes in Yaoundé generally have paint on the interior and/or exterior that can be contributing to the observed BLLs. Most of the development in the city took place after the 1960s and the average age of the homes of participants in the study was 29 years. As older homes generally have more paint deterioration and require more renovations, it is possible that exposure opportunities may increase as the housing stock in Yaoundé ages.

Our questionnaire inquired about other potential sources of lead exposure including parents’ occupation, hobbies, smoking habits, type of plumbing, paint conditions, daycare facilities, stove usage, and housing conditions. However, as we did not conduct any home investigations, environmental testing, or even home visits this limited our ability to evaluate these exposure sources. As noted, the lack of a reported response by many of the study volunteers to interviewers’ questions seeking these assessments also limited our interpretation of the data. These were some of the limitations in the study design.

Other sources of lead have been previously identified in Cameroon. Pregnant women often consume calabash clay that is sold in markets as a remedy for morning sickness and for other assumed beneficial properties. A small survey of these clays purchased by the authors in markets in Yaoundé in 2010 had concentrations of lead from 11 to 21 ppm (unpublished). Other samples from the region purchased in the U.S. contained 6.6 ppm (12). An earlier study of Calabash clay samples purchased in the United Kingdom were tested using Energy dispersive X-ray fluorescence and found to contain a mean concentrations of lead of 40 ppm (13). To the extent that mothers pass on their lead exposures to the developing fetus, this source of exposure can be one contributing factor in BLLs observed.

However, given the inconsistent nature of the Calabash clays and the likely recall bias inherent in asking about the frequency and quantity consumed during pregnancy, we did not include the consumption of clays in the questionnaire. We also made no attempt to include household income or other measures of socioeconomic status. Given that we only interviewed a single parent or caretaker for each participating child, we believed that responses to any questions on household income could be inaccurate and difficult to interpret.

A study in Cameroon of heavy metals found in common foods after typical preparation indicated that significant concentrations of lead are present particularly in condiments/spices and tubers and starches. Estimated mean lead exposures from food in Cameroon were 0.963 μg kg^−1^ body weight day^−1^ in contrast to similar study in France finding 0.2 μg kg^−1^ body weight day^−1^ (2).

Although the source of the lead in foods tested in that study were not traced to determine potential sources of contamination, commonly used aluminum cookware in Cameroon is known to contain significant concentrations of lead. Weidenhamer et al. reported significant levels of lead up to 260 μg per serving in leachate after simulated normal cooking conditions with cookware from Cameroon (1). These pots are made in small artisanal workshops from recycled metal from a range of sources and were obtained from markets in Yaoundé and other cities in Cameroon. This study also demonstrated that this type of aluminum cookware that is not anodized can release significant concentrations of lead although it generally contains less than 1,000 parts per million lead based on X-ray fluorescence testing.

Lead exposures from these various sources, and the resulting BLLs reported in this study, are having a direct impact on economic development in Cameroon. It is estimated that lead exposure costs Cameroon approximately $2.52 billion USD annually. This cost is about three times, the amount Cameroon receives in annual development aid ($ 852,290,000 USD) (14).

This study provides additional evidence that elevated BLLs remain persistent in sub-Saharan Africa. BLLs in Cameroon more than a decade after the ban on lead in gasoline are more than sixfold higher than the geometric mean BLL reported in the U.S. and more than fivefold higher than those reported in France (15, 16).

Cameroon must develop the capacity to test BLLs in order to better assess exposures and monitor progress in reducing levels going forward. This study points to the need to conduct ongoing surveillance and to enable medical practitioners to evaluate a patient’s BLL as appropriate.

Many sources of lead exposure remain unregulated in Cameroon including the use of lead in paints and in consumer products such as aluminum cookware. Industrial emissions may also be a contributing source. Additional action is needed to address this avoidable public health threat through effective regulations and standards to control these sources of exposure. Environmental assessments are needed to better determine potential exposure from soil, dust, water, and other environmental sources. In addition, more outreach and education, particularly to populations at risk of lead poisoning, are needed to facilitate risk reduction measures.

## Ethics Statement

This study protocol was granted ethical clearance on July 31, 2015, by the Comite National d’Ethique de la Recherche pour La Santé Humaine in Cameroon. This study was carried out in accordance with the recommendations of the Committee with written informed consent obtained from all subjects. Researchers briefed parents or guardians accompanying each child who attended the blood lead testing clinics on study protocols and were asked to sign a consent form to allow their children to participate. This study presents no more than minimal risk of harm to subjects and involves no procedures for which written consent is normally required outside of the research context.

## Author Contributions

FM was the Principal Investigator and obtained the ethics approval, designed the questionnaire, conducted the research, and reviewed the manuscript for the publication. DC and PA conducted the field research and testing and made substantial contributions to the collection of the data. GK and A-CB reviewed the manuscript and offered critical intellectual content revisions. PG was involved in the design of the study and wrote the primary draft of the manuscript.

## Conflict of Interest Statement

The authors declare that the research was conducted in the absence of any commercial or financial relationships that could be construed as a potential conflict of interest.

## References

[1] Weidenhamer JD Kobunski PA Kuepouo G Corbin RW Gottesfeld P. Lead exposure from aluminum cookware in Cameroon. Sci Total Environ (2014) 496:339–47. 10.1016/j.scitotenv.2014.07.016 25087065

[2] Gimou M-M Pouillot R Charrondiere UR Noël L Guérin T Leblanc JC Dietary exposure and health risk assessment for 14 toxic and essential trace elements in Yaoundé: the Cameroonian total diet study. Food Addit Contam Part A Chem Anal Control Expo Risk Assess (2014) 31(6):1064–80. 10.1080/19440049.2014.909953 24684161

[3] Walsh CM van Rooyen FC. Household food security and hunger in rural and Urban Communities in the Free State Province, South Africa. Ecol Food Nutr (2015) 54(2):118–37. 10.1080/03670244.2014.964230 25551521

[4] Hodge J Nielsen J Dignam T Brown MJ Small Area Surveillance to Estimate Prevalence of Childhood Blood and Environmental Lead Levels. (2016). Available from: https://www.cdc.gov/nceh/lead/BLL_PrevalenceStudy_TrainingManual_Final_508.pdf

[5] Ugwuja EI Ogbu IS Umeaku EA Otuu FC. Blood lead levels in children attending a tertiary teaching hospital in Enugu, South-Eastern Nigeria. Paediatr Int Child Health (2014) 34(3):216–9. 10.1179/2046905514Y.0000000118 24804562

[6] Tuakuila J Martin K Honoré M Gerard M Blood lead levels in children after phase-out of leaded gasoline in Kinshasa, the capital of Democratic Republic of Congo (DRC). Arch Public Health (2013) 71:5 10.1186/0778-7367-71-5 23556999 PMC3620025

[7] Naicker N Mathee A Barnes B. A follow-up cross-sectional study of environmental lead exposure in early childhood in urban South Africa. S Afr Med J (2013) 103(12):935–8. 10.7196/samj.7157 24300633

[8] Bodeau-Livinec F Glorennec P Cot M Dumas P Durand S Massougbodji A Elevated blood lead levels in infants and mothers in Benin and potential sources of exposure. Int J Environ Res Public Health (2016) 13(3):40–8. 10.3390/ijerph13030316 26978384 PMC4808979

[9] Ettinger AS Roy A Amarasiriwardena CJ Smith D Lupoli N Mercado-García A Maternal blood, plasma, and breast milk lead: lactational transfer and contribution to infant exposure. Environ Health Perspect (2014) 122:87–92. 10.1289/ehp.1307187 24184948 PMC3888576

[10] UN Environment Programme. Status of Leaded Gasoline Phase-Out in Sub-Saharan Africa. (2016). Available from: http://www.unep.org/transport/PCFV/PDF/Maps_Matrices/Africa/matrix/MatrixSSAfrica_Leadphaseout_august2011.pdf

[11] Gottesfeld P Kuepouo G Tetsopgang S Durand K. Lead concentrations and labeling of new paint in Cameroon. J Occup Environ Hyg (2013) 10(5):243–9. 10.1080/15459624.2013.768934 23472856

[12] U.S. Centers for Disease Control and Prevention (CDC). MMWR (2012). Available from: https://www.cdc.gov/mmwr/preview/mmwrhtml/mm6133a1.htm

[13] Dean JR Deary ME Gbefa BK Scott WC. Characterisation and analysis of persistent organic pollutants and major, minor and trace elements in Calabash chalk. Chemosphere (2004) 57(1):21–5. 10.1016/j.chemosphere.2004.05.023 15288195

[14] New York University School of Medicine, Department of Pediatrics (NYU). (2016). Economic Costs of Childhood Lead Exposure in Low-and Middle-Income Countries. Available from: http://nyulmc.org/pediatricleadexposure

[15] U.S. Centers for Disease Control and Prevention (CDC). Blood lead levels in children aged 1–5 years-United States, 1999–2010. MMWR Morb Mortal Wkly Rep (2013) 62(13):245–8. 23552225 PMC4605011

[16] Etchevers A Bretin P Lecoffre C Bidondo ML Le Strat Y Glorennec P Blood lead levels and risk factors in young children in France, 2008–2009. Int J Hyg Environ Health (2014) 217(4):528–37. 10.1016/j.ijheh.2013.10.002 24262290

